# Green Efficient One-Pot Synthesis and Separation of Nitrones in Water Assisted by a Self-Assembled Nanoreactor

**DOI:** 10.3390/ijms23010236

**Published:** 2021-12-26

**Authors:** Vincenzo Patamia, Giuseppe Floresta, Venerando Pistarà, Antonio Rescifina

**Affiliations:** 1 Dipartimento di Scienze Chimiche, Università di Catania, Viale A. Doria 6, 95125 Catania, Italy; vincenzo.patamia@unict.it; 2 Dipartimento di Scienze del Farmaco e della Salute, Università di Catania, Viale A. Doria 6, 95125 Catania, Italy; giuseppe.floresta@kcl.ac.uk (G.F.); vpistara@unict.it (V.P.)

**Keywords:** supramolecular chemistry, nanoconfined reactions, DOSY experiments

## Abstract

This article reports an alternative method for preparing nitrones using a tetrahedral capsule as a nanoreactor in water. Using the hydrophobic cavity of the capsule allowed us to reduce the reaction times and easily separate the nitrones from the reaction mixture, obtaining reaction yields equal or comparable to those obtained with the methods already reported. Furthermore, at the basis of this methodology, there is an eco-friendly approach carried out that can certainly be extended to other synthesis methods for the preparation of other substrates by exploiting various types of macrocyclic hosts, suitably designed and widely used in supramolecular chemistry.

## 1. Introduction

**Figure 1:**
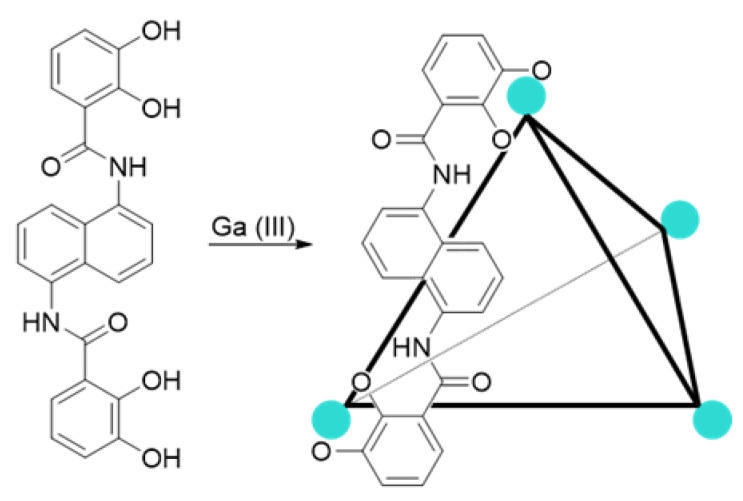
Tetrahedral capsule.

One of the purposes of a supramolecular chemist is to mimic biological systems, where complex reactions with high noncatalyzed energy barriers proceed effectively with excellent selectivity. Effective control of enzymes on substrates is achieved by isolating substrates in the active sites. Therefore, it is evident to make parallelisms between the active enzyme sites and the microenvironments of the supramolecular hosts, since both are based on molecular recognition, substrate isolation, and conformational control. Nowadays, a wide range of supramolecular systems that try to mimic the work carried out by enzymes have been reported in the literature, and various types of building blocks are being studied, producing a wide range of cavities that can be used for catalysis [1,2,3,4,5,6,7]. This work aims to use the already reported tetrahedral capsule **1** (Figure 1) as a nanoconfining catalyst in water.

The supramolecular capsule **1**, developed by the Raymond group, was employed to catalyze cyclization reactions, stabilize some substrates in water, and more [1]. In this study, the hydrophobic cavity of the tetrahedral capsule has been exploited to catalyze a dehydration reaction for the synthesis in water of nitrones, which are very useful substrates for the synthesis of biologically important nitrogen compounds. They also have relevant biological applications, e.g., as radical traps, thanks to the stabilization of nitroxide radicals in vitro and in vivo. In order to achieve this stabilization, the nitrone reacts with a free radical to form a relatively stable derivative called a spin-adduct that then becomes inactivated and unable to interfere with biochemical processes and damage cell tissues [8].

Nitrones are 1,3-dipoles of the allyl anion type, used to synthesize isoxazolidines by reaction with dipolarophiles, such as alkenes and acetylenes [9,10]. Generally, nitrones are obtained in dry conditions and purified employing a chromatographic column [11]. The tetrahedral capsule as a confining nanoreactor allowed us to synthesize these substrates using water as a solvent and separate them by extractions with organic solvents. The supramolecular system is self-assembled by six bis-bidentate ligands and four metal ions to form a cage with metal atoms at the tetrahedron vertices. As demonstrated by the crystallographically obtained geometric parameters and its T symmetry, the tetrahedron has ligands that cross the six edges. The ligand is *N*,*N’*-(naphthalene-1,5-diyl)bis(2,3-dihydroxybenzamide), consisting of a naphthalene core and two catechol groups capable of complexing the Ga^3+^ that constitute the vertices of the tetrahedron (Figure 1) [1].

The stoichiometry of the capsule is [K_12_(Ga_4_L_6_)], and since each Ga(III)-triscatecholate has a formal trianionic charge, it is overall dodecanionic. By this high charge, the host is soluble in water and polar organic solvents; it is within the nanoscale regime in size, measuring about 13 Å from vertex to vertex [1].

## 2. Results and Discussion

Capsule **1** was synthesized according to the method reported in the literature [12], employing 2,3-dimethoxybenzoic acid and naphthalene-1,5-diamine as building blocks for the ligand synthesis and gallium(III) acetylacetonate in water for the assembly; all details are reported in Scheme S1. The correct assembly has been verified employing tetraethylammonium chloride salt [13] (Scheme S2). The DOSY experiment, in which the signals of the ammonium salt result in shielding of negative ppm values and present the same diffusion coefficient of the capsule signals, underlines the formation of the complex (Figure S1).

It was possible to synthesize six different nitrones, starting from aldehydes and ketones, taking advantage of the capsule’s hydrophobic cavity. The advantage of using the capsule as a nanoreactor was the easy separation of the nitrones obtained by extraction with a green classified organic solvent [14,15]. On the contrary, the traditional methodologies for synthesizing these substrates foresee the use of chromatographic separation [11]. Furthermore, for some substrates, the products were obtained with shorter reaction times and were in higher yields than those reported in the literature [16].

To optimize the reaction conditions, the synthesis of *N*-methyl-1-phenylmethanimine oxide (**4a**) was used as a model reaction (Table 1). Firstly, we carried out the reaction in water without the capsule, obtaining only traces of the product (Table 1, entry 1), as already verified in other works in the literature; being a dehydration reaction, the presence of water as the reaction solvent does not allow the formation of the nitrone [2,17]. The amounts of benzaldehyde (**1**) and *N*-methylhydroxylamine hydrochloride (**3**) were varied. Subsequently, even the ratio of the capsule was varied from 1 eq. to 0.1 eq. Using a catalytic amount of capsule (Table 1, entry 7) allowed us to obtain good reaction yields in shorter times [11]. To further verify that the reaction takes place inside the hydrophobic cavity of the capsule, we performed the reaction in the presence of the highly competitive guest tetraethylammonium chloride (*K*_a_ = 19.6 × 10^3^ M^−1^ in D_2_O) [18]. With an equimolar amount of salt with respect to the capsule (0.2 eq.), the reaction yield was lowered to 40% (Table 1, entry 9), whereas, doubling the equivalents (0.4 eq.), we achieved only traces of the nitrone product (Table 1, entry 10). These data underline that ammonium salt, having a high affinity with the capsule cavity, prevents the reagents from entering the hydrophobic cavity, precluding the reaction course.

At the same time, to verify a possible product inhibition, we performed two other experiments employing both one and two equivalents of nitrone **4a** with respect to the capsule (Table 1, entries 11 and 12) at the start of the reaction. Effectively, the product causes the inhibition of the reaction, suggesting that nitrone could be more affinal toward the capsule than the reagents. Additionally, the total amount of the nitrone present at the end of each reaction result was almost unaltered and corresponded to the yield obtained in the model reaction (Table 1, entry 7).

With the optimized reaction conditions, the catalytic capacity of the capsule was tested using five other substrates, including aldehydes and ketones. Table 2 shows the reaction times and the yields obtained with respect to those reported in the literature. In addition, a never-reported nitrone was synthesized from acetaldehyde (Table 2, entry 2).

**Figure 2:**
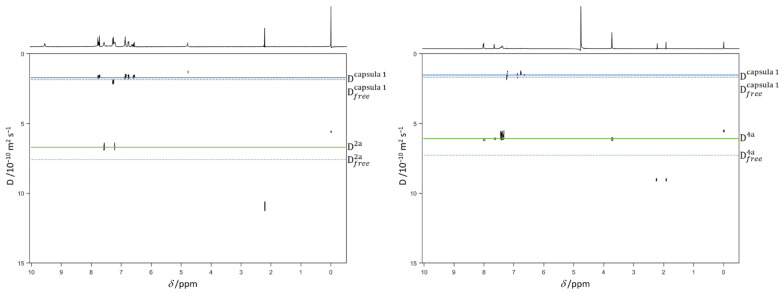
(**Left**) 2D DOSY-NMR measurements on the D_2_O solution of **2a** (0.025 M) and capsule **1** (0.005 M) mixture. (**Right**) 2D DOSY-NMR measurements on the D_2_O solution of **4a** (0.025 M) and capsule **1** (0.005 M) mixture. The horizontal axis represents the chemical shifts, whereas the vertical axis the diffusion coefficients; the black spots are the resonances of the water solution of the inclusion complex spread in the second dimension, according to their measured diffusion coefficient.

To examine the inclusion of the studied substrates (**2a** and **4a**) inside the catalytic cavity and estimate the association constant *K*_a_ at the same time, we performed a series of DOSY experiments on free capsule **1**, compounds **2a** and **4a**, and the **2a**@**1** and **4a**@**1**complexes (Figure 2 and Figures S15–S20). Since the systems investigated in this study were under fast equilibrium between free and complexed states on the NMR time scale, the observed diffusion coefficient (D_obs_) measured in the experiment was the weighted average of those of the free and complexed molecules [22]:

D_obs_ = *x*_HG_ D_bound_ + (1 − *x*_HG_) D_free_, (1)

where D_free_ and D_bound_ are the diffusion coefficients of free and bound guest molecules, respectively, whereas *x*_HG_ refers to the molar fraction of the host–guest (HG) complex.

The solid lines green and purple in Figure 2 represent the diffusion observed for **2a** or **4a** $\left(D_{\mathrm{obs}}^{2a\;\mathrm{or}\;4a}\right)$ and capsule **1** $\left(D_{\mathrm{obs}}^{\mathrm{capsule}\;1}\right)$, respectively, while the dashed lines are the diffusion coefficients of free **2a** or **4a** $\left(D_{\mathrm{free}}^{2a\;\mathrm{or}\;4a}\right)$ and free capsule **1**$\;\left(D_{\mathrm{free}}^{\mathrm{capsule}\;1}\right)$.

The quantitative estimation of the complex formation constant is based on the degree to which the solid lines (complexed molecules) are displaced from their corresponding dashed (free molecules).

The results demonstrate that the diffusion coefficients for both the molecules **2a** and **4a** diminished from 7.42 to 6.82 × 10^−10^ m^2^/s and from 6.77 to 6.00 × 10^−10^ m^2^/s for molecules **2a** and **4a**, respectively (Table 3), and this is indicative of a complexation within the cavity of molecule **1**.

The association constant *K*_a_ can be calculated exploiting a single-point procedure on the assumption of the known *x*_HG_ (**2a** or **4a**) (Equation (2)) [23,24]. This procedure assumes that the diffusion coefficient of the host–guest complex (**2a**@**1** and **4a**@**1** complexes) is the same as that of the host molecule (capsule **1**). This is because the host molecule is usually very much larger than the guest, so it seems reasonable to assume that the diffusion coefficient of the host–guest complex is the same as that of the host molecule (a measurable quantity).

*K*_a_ = *x*_HG_/[(1 − *x*_HG_) ([H] − *x*_HG_ [G])], (2)

In Equation (2), [H] and [G] are the total concentrations of the host and guest, respectively. The diffusion coefficients (D) measured in D_2_O and the respective association constants are reported in Table 3. As pointed out by the DOSY experiments, both the molecules **2a** and **4a** can be included inside the hydrophobic cavity of capsule **1**. Moreover, product **4a** is able to achieve a stronger interaction with the host molecule; this agrees with the experimental result that the higher the amount of capsule, the higher the yield of the reaction.

## 3. Materials and Methods

### 3.1. General Information

All the required chemicals were purchased from Merck (Merck KGaA, Darmstadt, Germany). The synthesis of the capsule building blocks and assembly were carried out as reported in the literature and subsequently characterized by NMR [12]. Precoated aluminum sheets (silica gel 60 F254, Merck) were used for thin-layer chromatography (TLC), and spots were visualized under UV light. Silica gel column chromatography was performed using silica gel 60–120-mesh sizes. ^1^H and ^13^C NMR spectra were recorded at 300 K on Varian UNITY Inova (Agilent, Santa Clara, CA, USA) using CDCl_3_ and D_2_O as the solvents at 500 MHz for ^1^H NMR and 125 MHz for ^13^C NMR. ^13^C spectra were ^1^H-decoupled, and the APT pulse sequence determined the multiplicities. Chemical shift (*δ*) values were given in ppm. Diffusion ordered spectroscopy (DOSY) experiments were performed using the DgcsteSL_cc (DOSY gradient compensated stimulated echo with spin-lock and convection compensation) HR-DOSY sequence. The pulsed gradient range amplitudes were 0.1067–0.5334 T m^−1^ at a diffusion time of 0.06 s. The processing program (DOSY macro in the Varian instrument) was run with the data transformed using fn = 32 K and lb = 0.3. All the experiments were acquired at 300 K.

### 3.2. General Procedure for the Synthesis of Nitrones ***4a**–**e*** with Capsule ***1***

Nitrones were synthesized from commercially available aldehydes and ketones (1.0 eq.), *N*-methylhydroxylamine hydrochloride (50.0 mg, 0.598 mmol, 1.0 eq.), NaHCO_3_ (50.3 mg, 0.598 mmol, 1.0 eq.), and capsule **1** (396.0 mg, 0.199 mmol, 0.2 eq.) in water (5 mL) at room temperature. All products were extracted with EtOAc (3 × 10 mL), collected without further purification, and brought to dryness at a reduced pressure. After the extractions, the aqueous phase, examined by tlc, did not show the presence of products **4**. The *E*/*Z* configuration of nitrones **4a**,**c**,**d** was assigned according to the literature data [11,19]. All products were characterized by ^1^H and ^13^C NMR.

#### 3.2.1. (*Z*)-*N*-Methyl-1-Phenylmethanimine Oxide (**4a**)

White solid. ^1^H NMR (500 MHz, Chloroform-*d*): *δ* = 8.35–8.11 (m, 2H), 7.69–7.33 (m, 4H), 3.87 (s, 3H); ^13^C NMR (126 MHz, Chloroform-*d*): *δ* = 134.42, 130.45, 130.33, 129.64, 128.93, 128.43, 128.14, 54.24.

#### 3.2.2. (*Z*)-Isomer of *N*-Methylethanimine Oxide (**4b**)

Light brown oil. ^1^H NMR (500 MHz, D_2_O): *δ* = 7.36 (q, *J* = 5.5 Hz, 1H), 3.71 (s, 3H), 2.04 (d, *J* = 5.8 Hz, 3H); ^13^C NMR (126 MHz, D_2_O): *δ* = 145.56, 50.82, 12.55.

#### 3.2.3. (*Z*)-*N*-Methylpropan-1-Imine Oxide (**4c**)

Colorless oil. ^1^H NMR (500 MHz, D_2_O): *δ* = 7.26 (t, *J* = 5.9 Hz, 1H), 3.67 (s, 3H), 2.48–2.41 (m, 2H), 1.12 (t, *J* = 7.7 Hz, 3H); ^13^C NMR (126 MHz, D_2_O): *δ* = 150.79, 50.83, 20.28, 8.65.

#### 3.2.4. (*E*)-2-Ethoxy-*N*-Methyl-2-Oxoethan-1-Imine Oxide (**4d**)

Yellow oil. ^1^H NMR (500 MHz, Chloroform-*d*): *δ* = 7.24 (s, 1H), 4.26 (dt, *J* = 14.3, 5.7 Hz, 3H), 4.18 (s, 3H), 1.32 (t, *J* = 7.1 Hz, 2H); ^13^C NMR (126 MHz, Chloroform-*d*): *δ* = 161.11, 127.70, 61.43, 52.26, 14.17.

#### 3.2.5. *N*-Methylcyclohexanimine Oxide (**4e**)

Light brown oil. ^1^H NMR (500 MHz, Chloroform-*d*): *δ* = 3.70 (s, 3H), 2.76 (t, *J* = 6.5 Hz, 1H), 2.71 (s, 1H), 2.64 (s, 1H), 2.47 (t, *J* = 6.4 Hz, 2H), 2.14 (d, *J* = 21.8 Hz, 1H), 1.69 (dt, *J* = 12.7, 6.3 Hz, 2H), 1.58 (ddd, *J* = 11.4, 7.2, 4.6 Hz, 2H); ^13^C NMR (126 MHz, Chloroform-*d*): *δ* = 150.11, 47.22, 29.99, 26.72, 25.25, 24.50.

#### 3.2.6. *N*-Methyl-1,1-Diphenylmethanimine Oxide (**4f**)

Pale yellow solid. ^1^H NMR (500 MHz, Chloroform-*d*): *δ* = 7.84–7.76 (m, 3H), 7.63–7.55 (m, 1H), 7.54–7.46 (m, 3H), 4.02 (s, 3H); ^13^C NMR (126 MHz, Chloroform-*d*): *δ* = 137.85, 132.59, 130.46, 130.20, 128.42, 42.46.

### 3.3. Classical Method for the Synthesis of Nitrones ***4a**–**e***

Into a 10-mL round flask containing dichloromethane (6 mL) at 0 °C under N_2_ atmosphere was added aldehyde or ketone (500 mg, 11.35 mmol, 5 eq.), *N*-methylhydroxylamine hydrochloride (189.60 mg, 2.27 mmol, 1 eq.), NaHCO_3_ (190.68 mg, 2.27 mmol, 1 eq.), and Na_2_SO_4_ (322.43 mg, 2.27 mmol, 1 eq.), and the solution was stirred for 1 h. The crude product was purified by silica gel column chromatography using dichloromethane:methanol (9:1) as the eluent to give the corresponding nitrones.

## 4. Conclusions

In conclusion, we reported the first example of nitrone synthesis using this tetrahedral capsule as a nanoreactor in water. We also synthesized a new nitrone, starting from acetaldehyde, using the capsule in water. The synthesis in a nanoconfined space allowed us to reduce the reaction times, in some cases, and easily separate the nitrones from the reaction mix, obtaining reaction yields equal to or comparable to those obtained with classical methodologies. The environmentally friendly approach underlying this work can be extended, in a relatively simple way, to other reactions that use various types of macrocyclic hosts suitably designed and widely used in supramolecular chemistry.

## Figures and Tables

**Table 1:** Optimization of the reaction conditions between aldehyde **1** and *N*-methylhydroxylamine **2** ^a^.

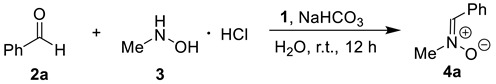				
Entry	2a (eq.)	3 (eq.)	Capsule 1 (eq.)	Yield (%)
1	1	1	—	Traces
2	1	1	1.0	74
3	2	1	1.0	73
4	3	1	1.0	75
5	1	3	1.0	73
6	1	1	0.5	71
7	1	1	0.2	70
8	1	1	0.1	58
9 ^b^	1	1	0.2	40
10 ^c^	1	1	0.2	Traces
11 ^d^	1	1	0.2	53
12 ^e^	1	1	0.2	29

^a^ All reactions were conducted with an equimolar amount of NaHCO_3_ with respect to **3**. ^b^ The reaction was carried out with an equimolar amount of tetraethylammonium chloride salt (0.2 eq.) with respect to the capsule. ^c^ The reaction was carried out with a double molar amount of tetramethylammonium chloride salt (0.4 eq.) with respect to the capsule. ^d^ The reaction was performed with an equimolar amount of **4a** (0.2 eq.) with respect to the capsule. ^e^ The reaction was performed with a double molar amount of **4a** (0.4 eq.) with respect to the capsule.

**Table 2:** Reaction times and yields obtained with the capsule and reported in the literature ^a^.

Entry	Substrate	Reaction Time (h)	Product	Yield (%)	Reaction Time Lit. (h)	Yield (%) Lit.
1	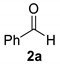	12	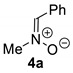	70	48	80 [11]
2	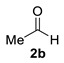	1	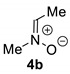	83	1	85 (this work) ^b^
3	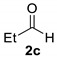	3	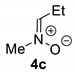	79	48	56 [11]
4	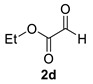	12	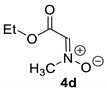	70	12	75 [19]
5	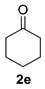	6	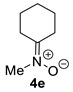	75	10 min	68 [20]
6	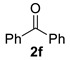	12	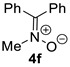	39	7	65 [21]

^a^ All the reactions were conducted in water at room temperature with 0.2 eq. of the capsule and 1.0 eq. of NaHCO_3_. ^b^ The *E*/*Z* configuration was assigned via 1D NOESY (see the ESI). All products were characterized by ^1^H and ^13^C NMR (see the ESI).

**Table 3:** Diffusion coefficients (D) of **1**, **2a**, **3**, **4a**, **2a**@**1**, **3**@**1**, and **4a**@**1**; the complex molar fraction; and the association constant (*K*_a_) of **2a**@**1**, **3**@**1**, and **4a**@**1**.

Compound	D (10^−10^ m^2^s^−1^)	Complex Molar Fraction (%)	*K*_a_ (M^−1^)
**1**	2.16	—	—
**2a**	7.42	—	—
**3**	9.80	—	—
**4a**	6.77	—	—
**2a**@**1**	6.86	10.64	50.88
**3**@**1**	9.42	4.90	10.55
**4a**@**1**	6.00	16.70	243.00

## Supplementary Materials

The following are available online at https://www.mdpi.com/article/10.3390/ijms23010236/s1.
