# Supplementary material for: Green Efficient One-Pot Synthesis and Separation of Nitrones in Water Assisted by a Self-Assembled Nanoreactor
Source: Int J Mol Sci. 2021 Dec 26;23(1):236. doi: 10.3390/ijms23010236 (PMC8745384; doi:10.3390/ijms23010236)
Supplement: Supplementary file 1 [file ijms-23-00236-s001.zip › ijms-1482720-supplementary.pdf]

Supporting Information

Green Efficient One-Pot Synthesis and Separation of Nitrones in Water Assisted by a Self-Assembled Nanoreactor

Vincenzo Patamia [1], Giuseppe Floresta [2], Venerando Pistarà [2] and Antonio Rescifina [2,*]

[1] Dipartimento di Scienze Chimiche, Università di Catania, Viale A. Doria 6, 95125 Catania, Italy; vincenzo.patamia@unict.it (V.P.)
[2] Dipartimento di Scienze del Farmaco e della Salute, Università di Catania, Viale A. Doria 6, 95125 Catania, Italy; giuseppe.floresta@kcl.ac.uk (G.F.); vpistara@unict.it (V.P.); arescifina@unict.it (A.R.)
* Correspondence: arescifina@unict.it (A.R.)

Table of contents

Scheme S1. Ligand synthesis procedure S3

Scheme S2. Capsule assembly with the tetraethylammonium salt S3

Figure S1. DOSY experiment of tetraethylammoniumsalt@capsule **1**. S4

Figure S2. ^{1}H NMR spectrum of **4a** S4

Figure S3. ^{13}C NMR spectrum of **4a** S5

Figure S4. ^{1}H NMR spectrum of **4b** S5

Figure S5. ^{13}C NMR spectrum of **4b** S6

Figure S6. 1D NOESY of **4b** S6

Figure S7. ^{1}H NMR spectrum of **4c** S7

Figure S8. ^{13}C NMR spectrum of **4c** S7

Figure S9. ^{1}H NMR spectrum of **4d** S8

Figure S10. ^{13}C NMR spectrum of **4d** S8

Figure S11. ^{1}H NMR spectrum of **4e** S9

Figure S12. ^{13}C NMR spectrum of **4e** S9

Figure S13. ^{1}H NMR spectrum of **4f** S10

Figure S14. ^{13}C NMR spectrum of **4f** S10

Figure S15. DOSY spectrum of **1** S11

Figure S16. DOSY spectrum of **2a** S11

Figure S17. DOSY spectrum of **3** S12

Figure S18. DOSY spectrum of **4a** S12

Figure S19. DOSY spectrum of **2a@1** S13

Figure S20. DOSY spectrum of **4a@1** S13

Figure S21. DOSY spectrum of **3@1** S14

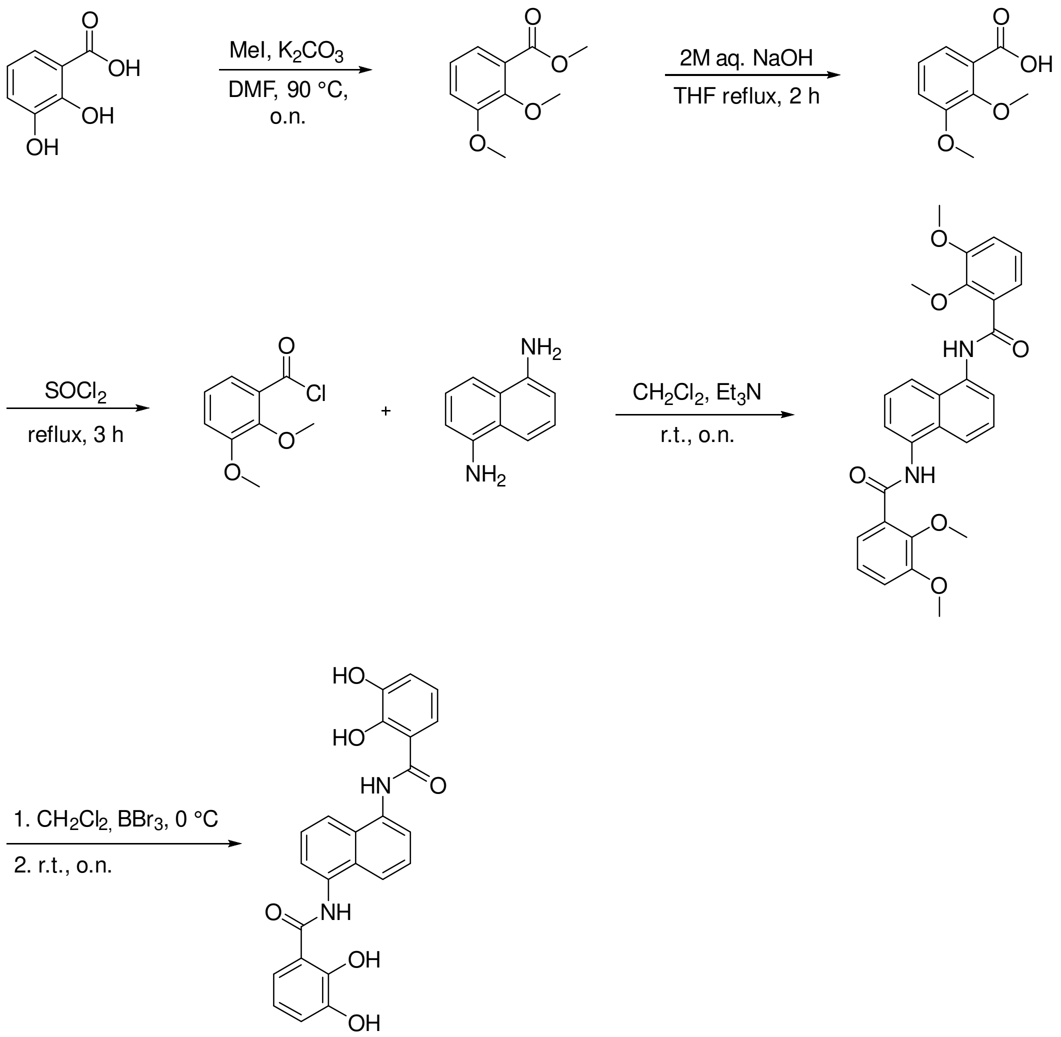

Scheme S1. Ligand synthesis procedure.

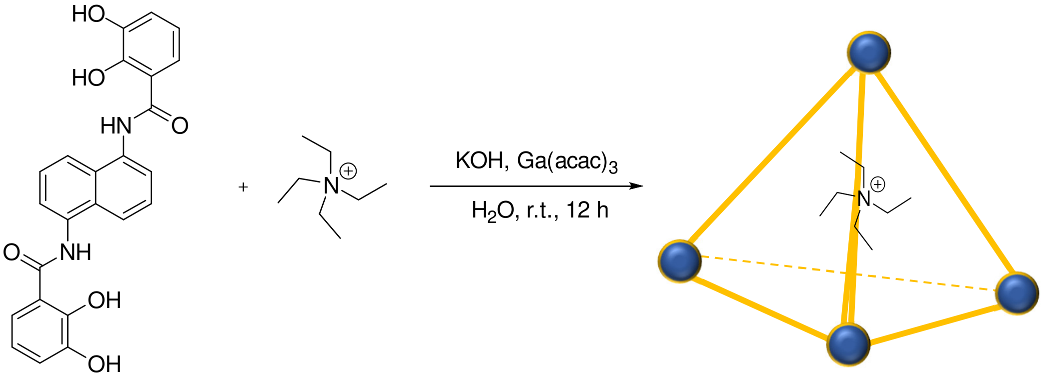

Scheme S2. Capsule assembly with the tetraethylammonium salt.

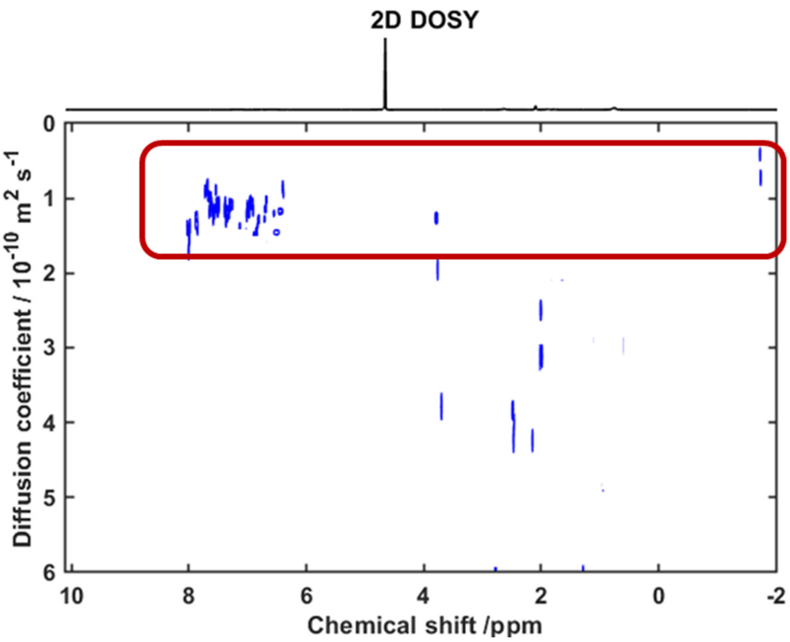

Figure S1. DOSY experiment of tetraethylammoniumsalt@capsule **1**.

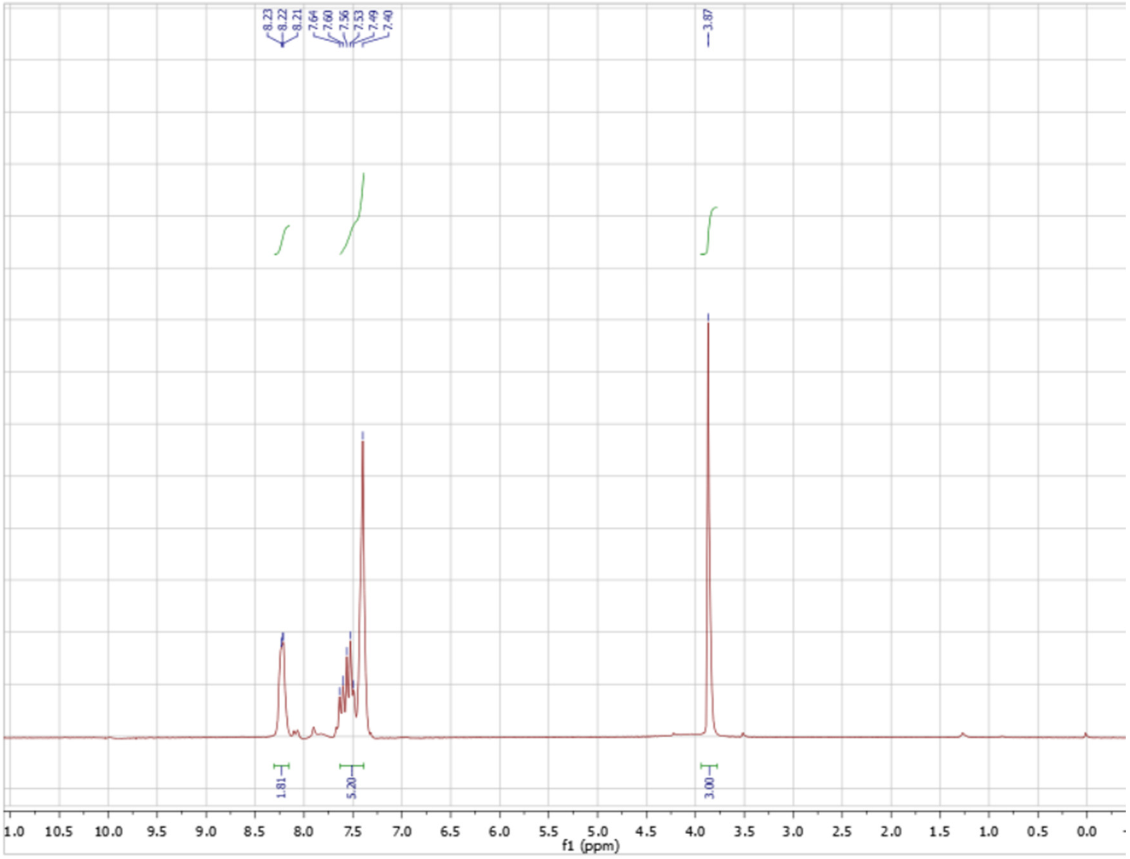

Figure S2. ^{1}H NMR **4a**.

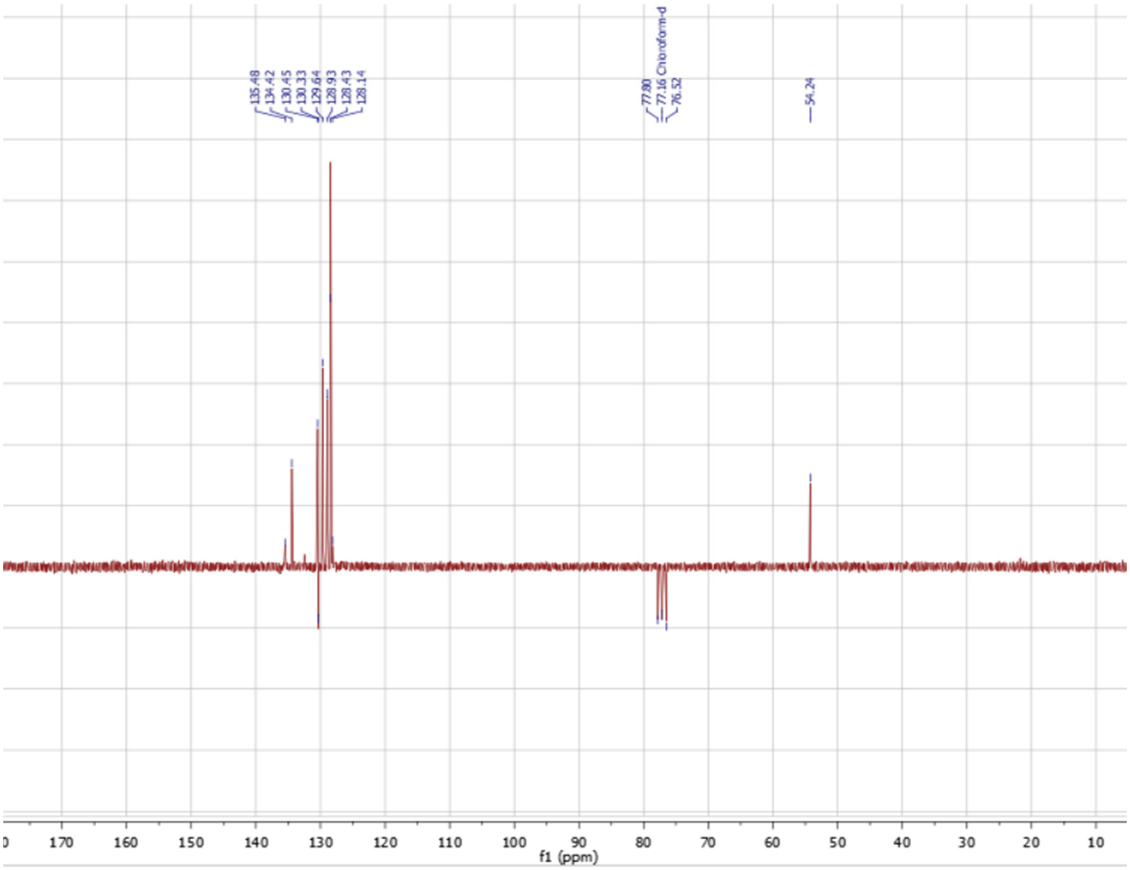

Figure S3. ^{13}C NMR **4a**.

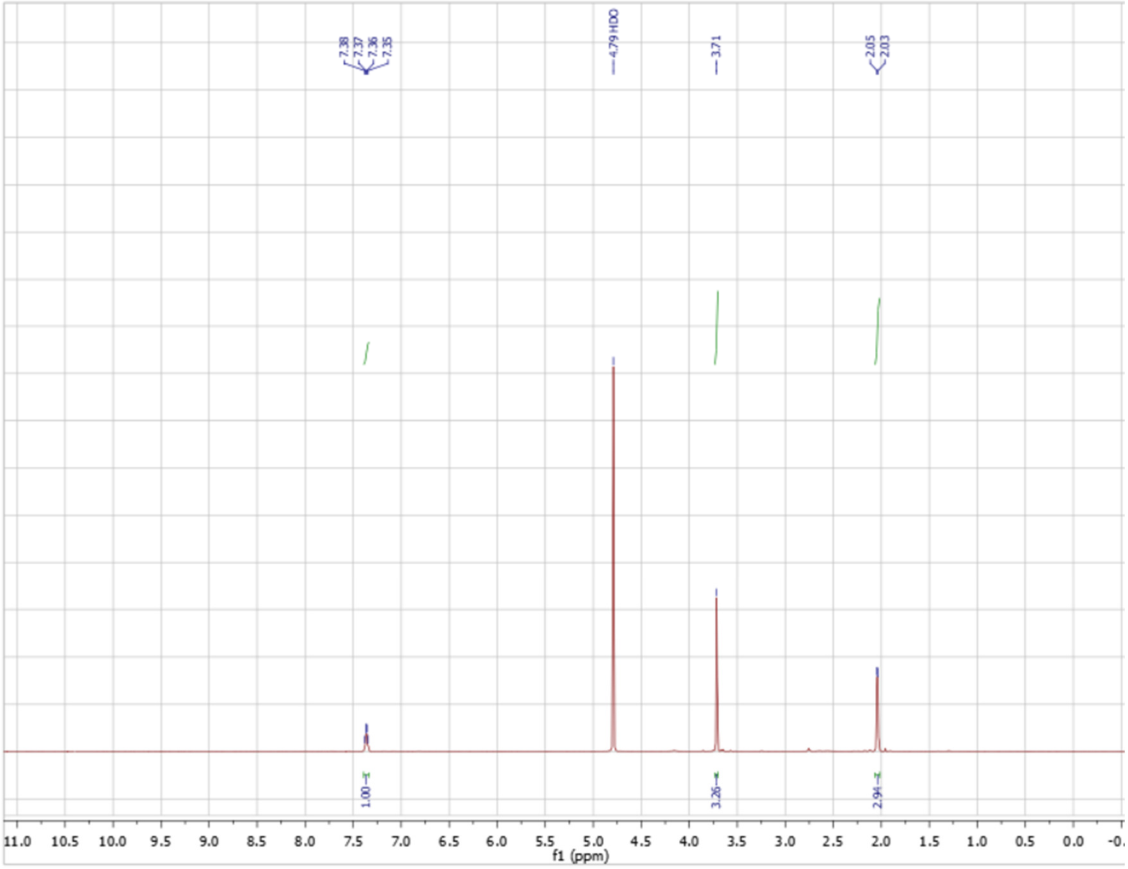

Figure S4. ^{1}H NMR **4b**.

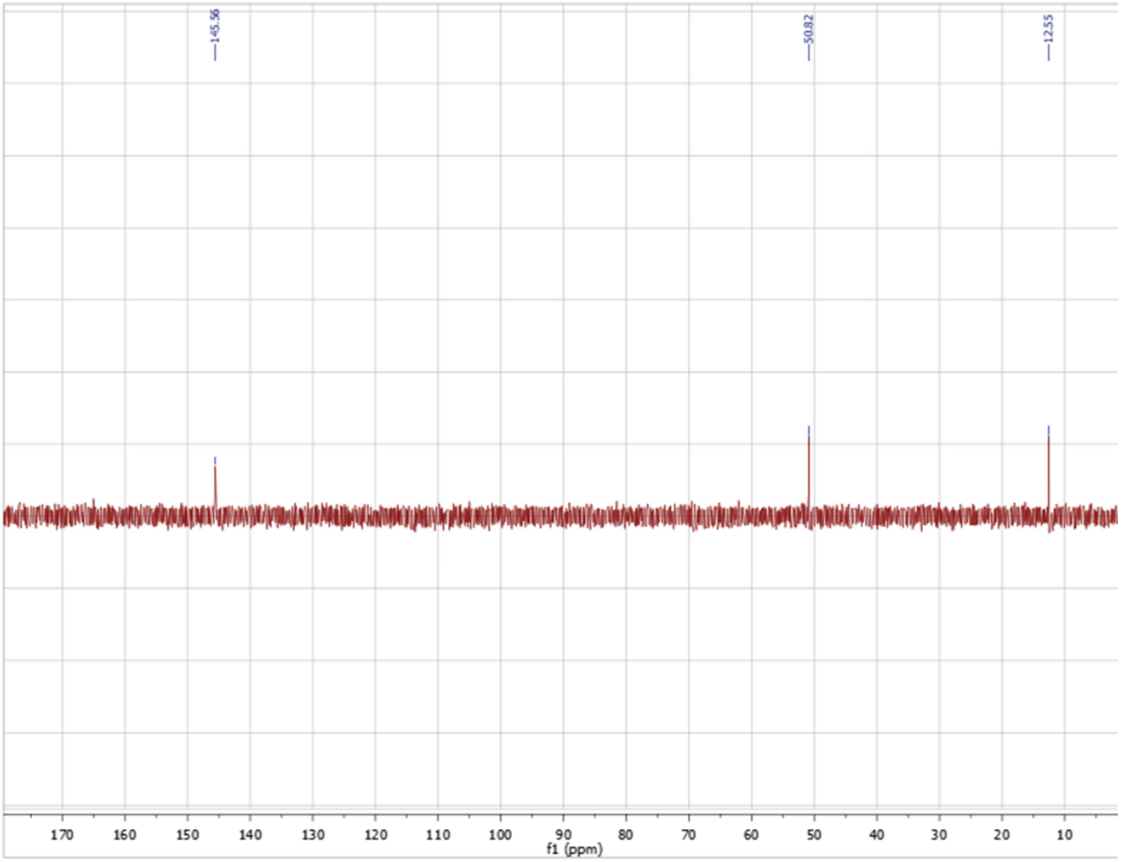

Figure S5. ^{13}C NMR **4b**.

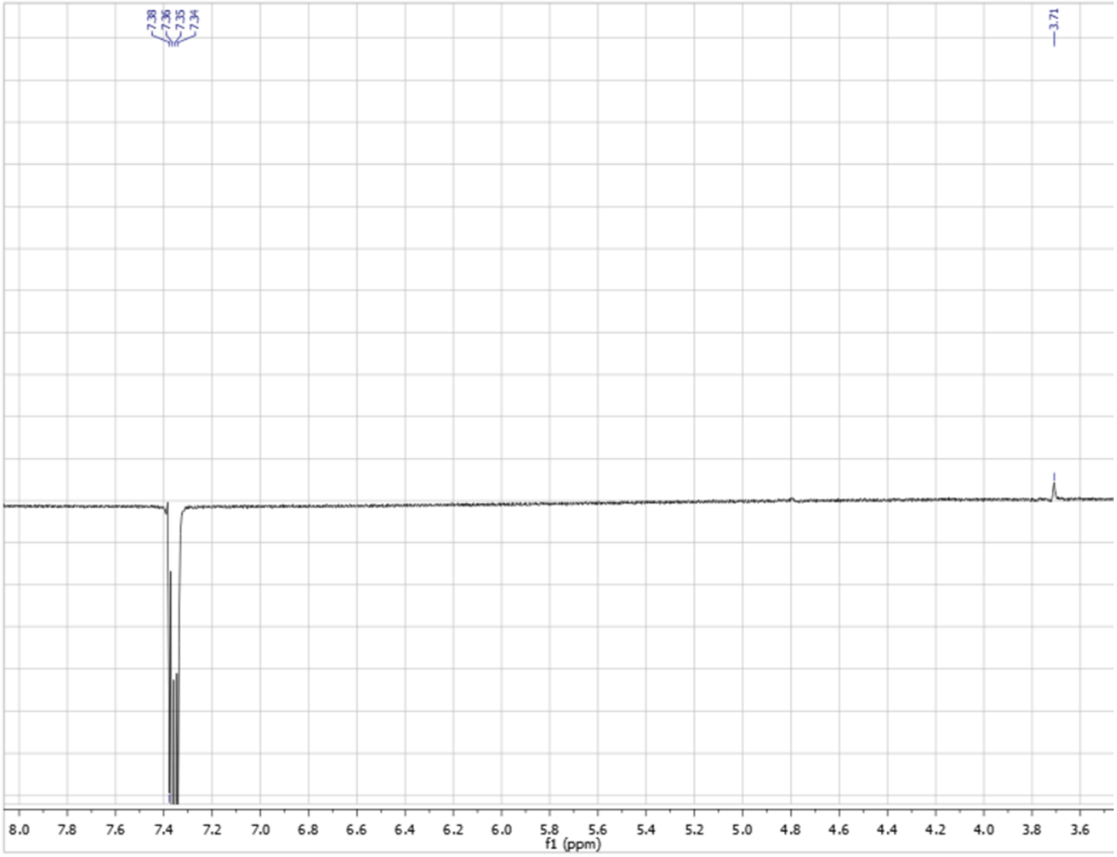

Figure S6. 1D NOESY **4b**.

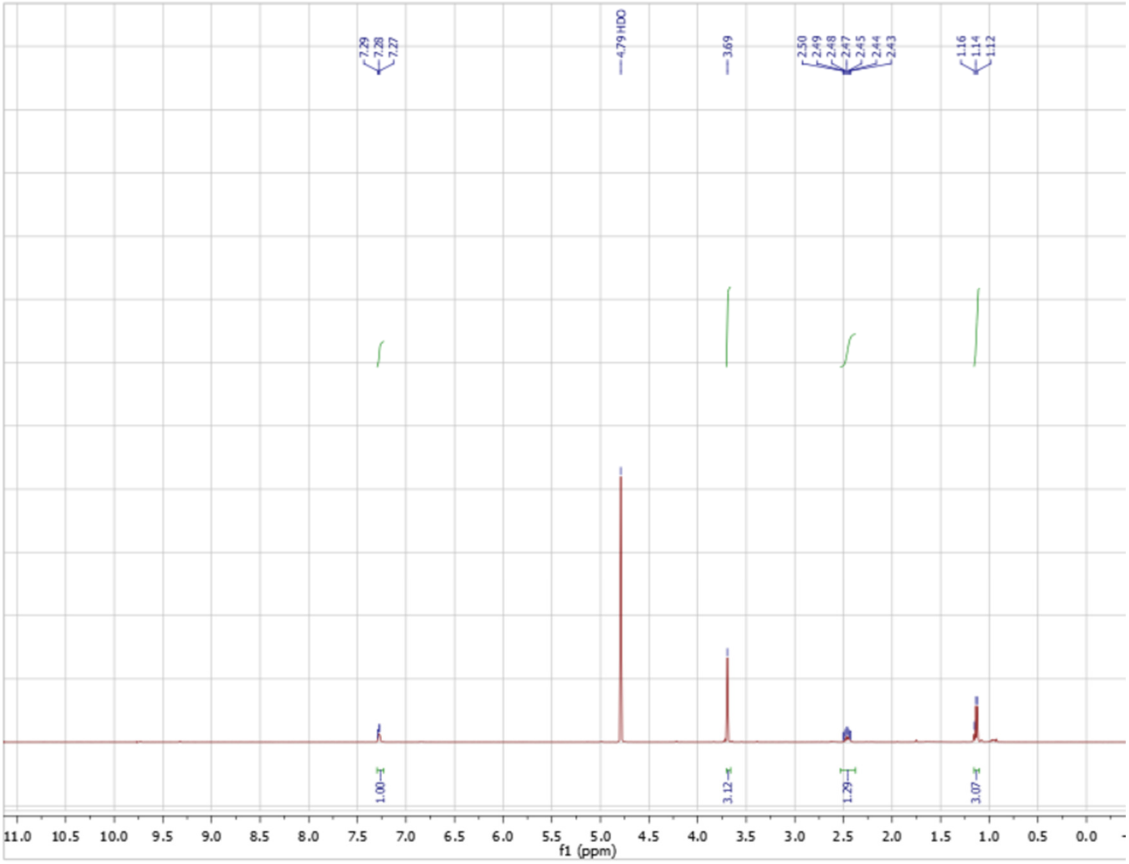

Figure S7. ^{1}H NMR **4c**.

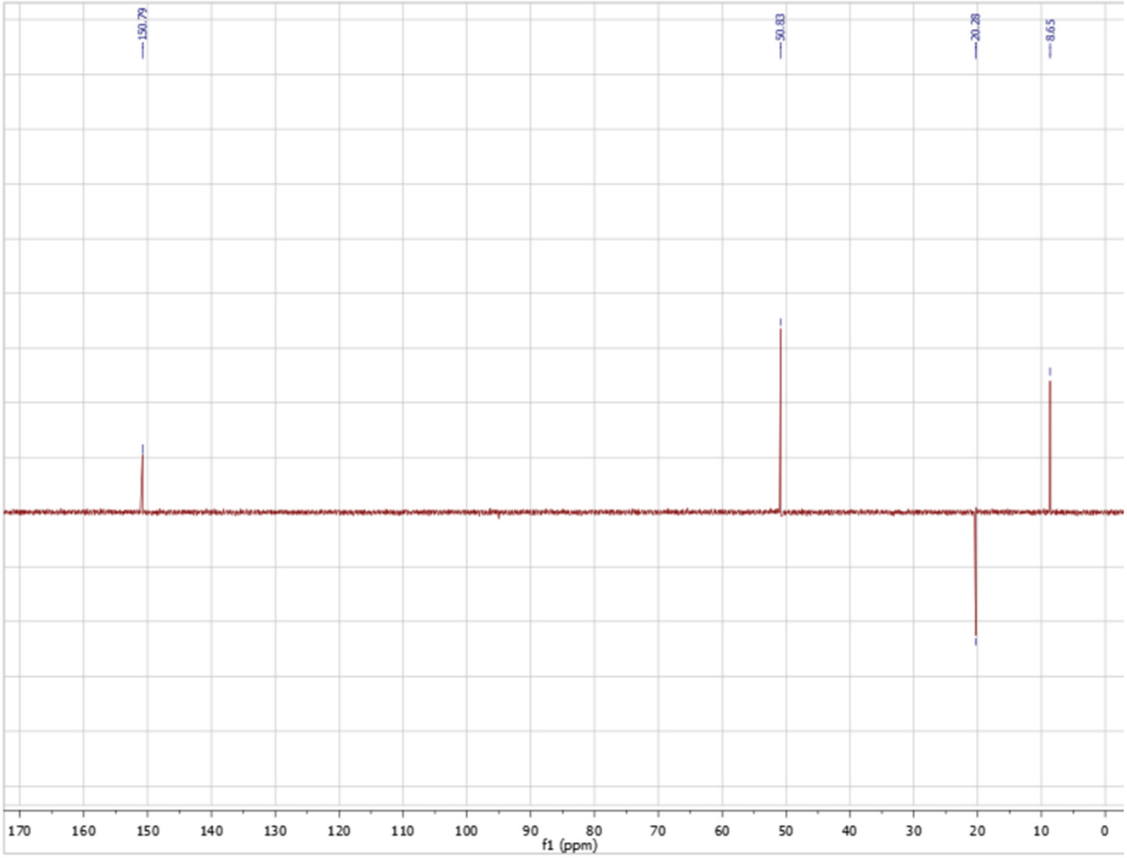

Figure S8. ^{13}C NMR **4c**.

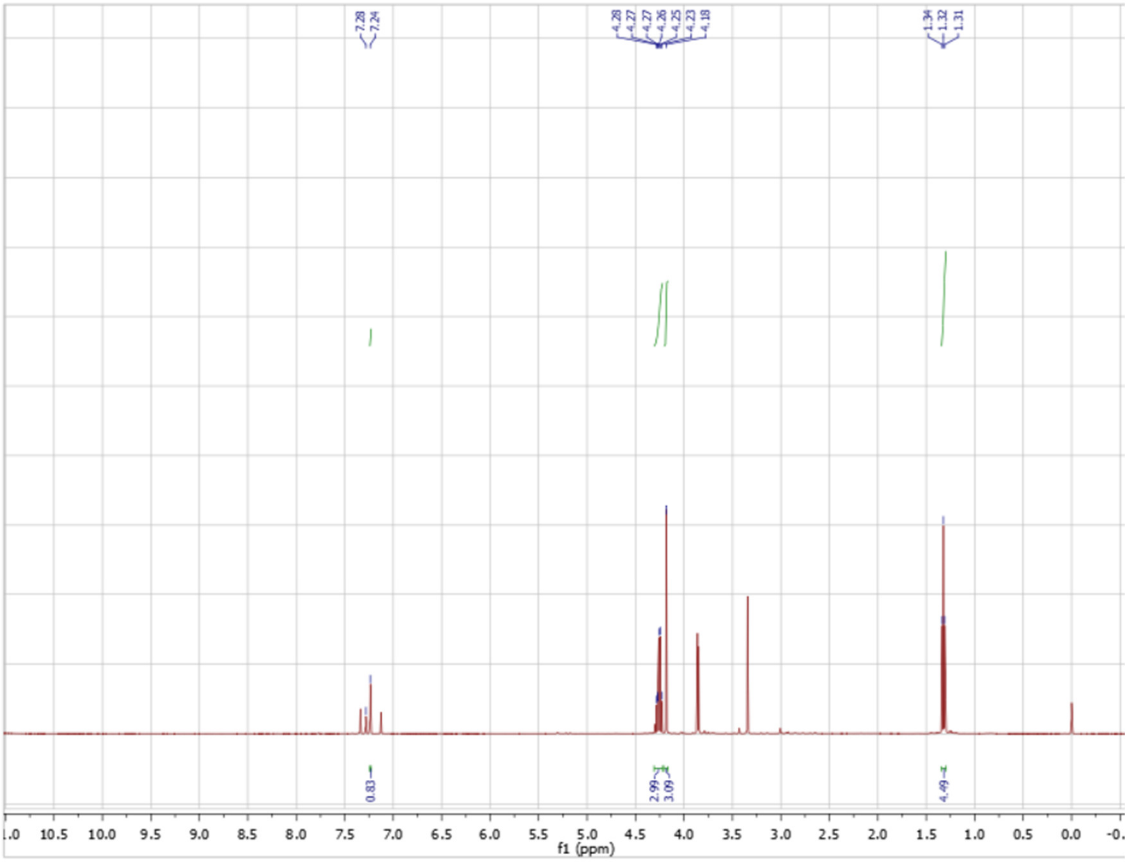

Figure S9. ^{1}H NMR **4d**.

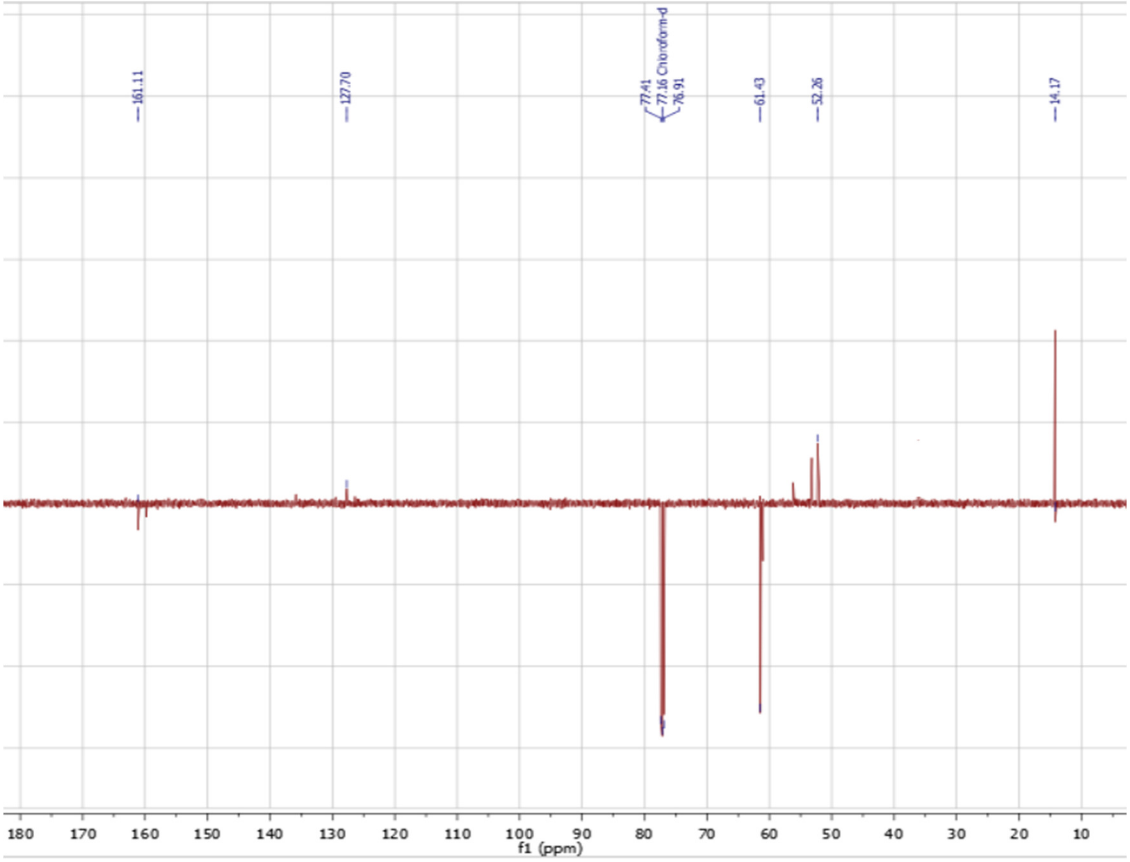

Figure S10. ^{13}C NMR **4d**.

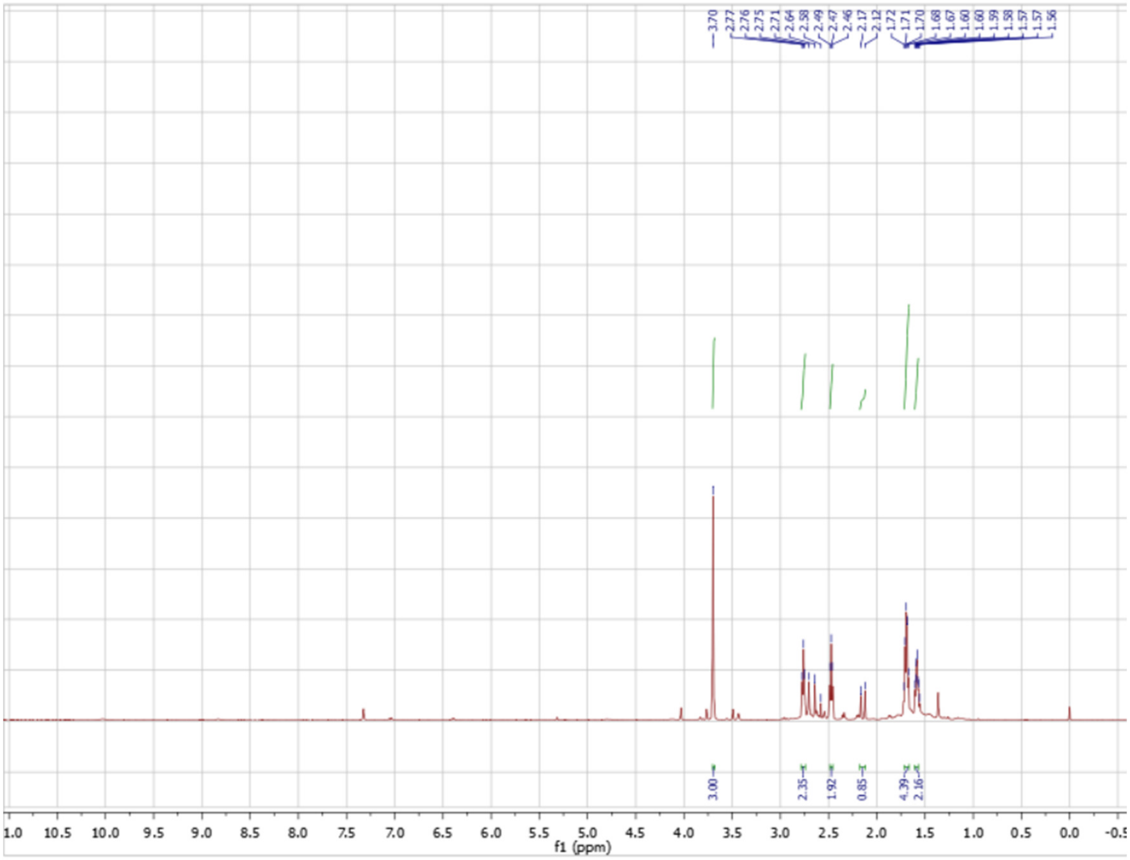

Figure S11. ^{1}H NMR **4e**.

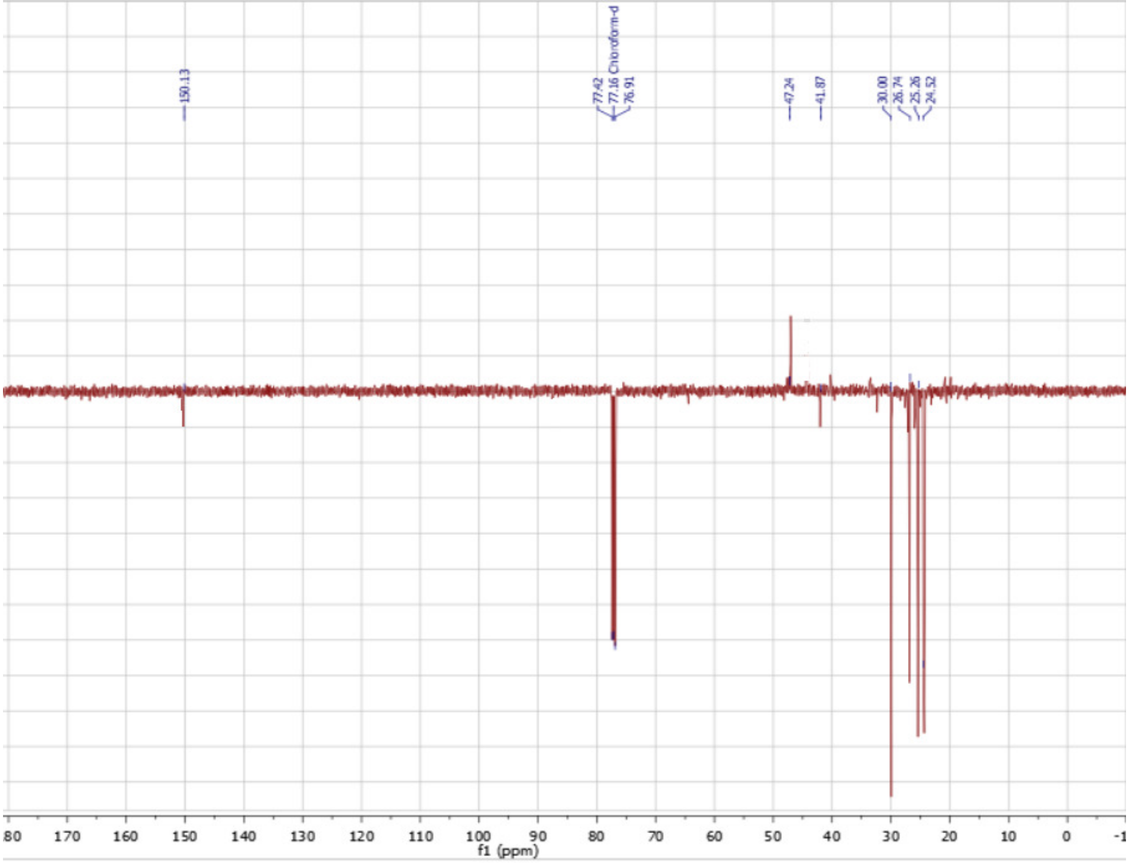

Figure S12. ^{13}C NMR **4e**.

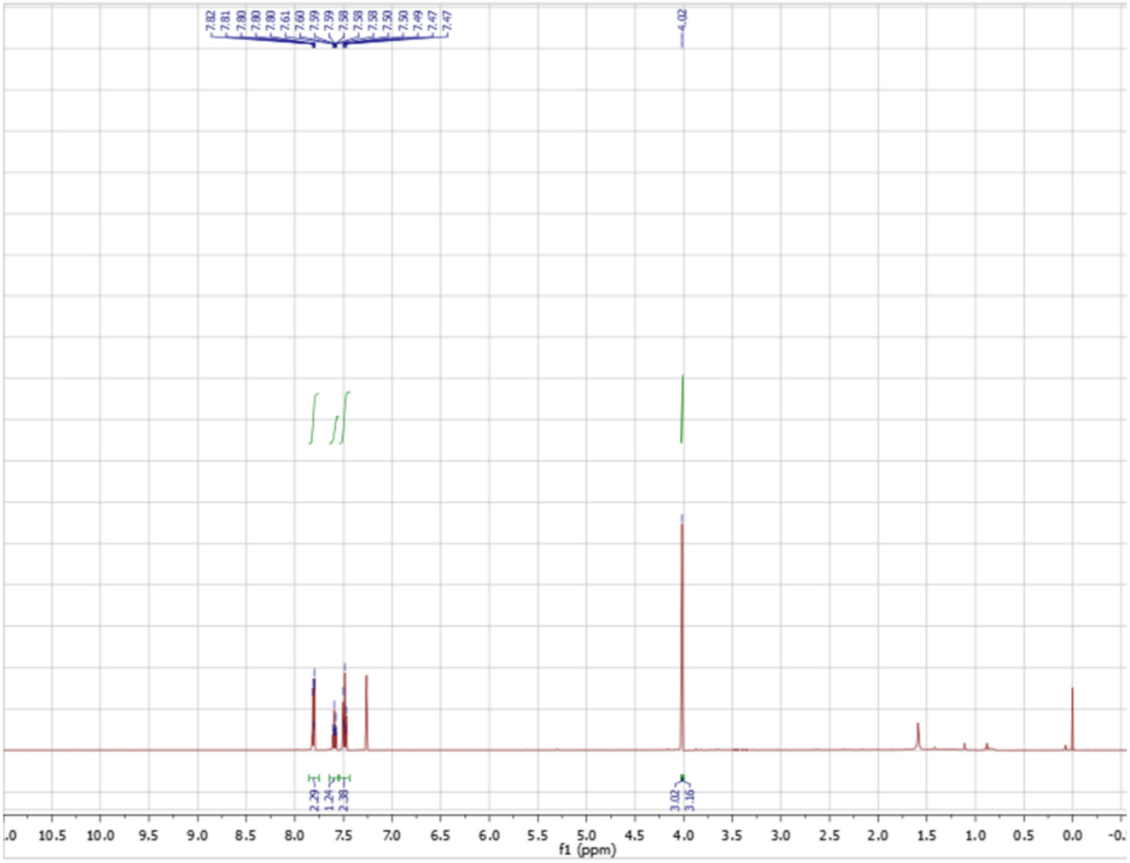

Figure S13. ^{1}H NMR **4f**.

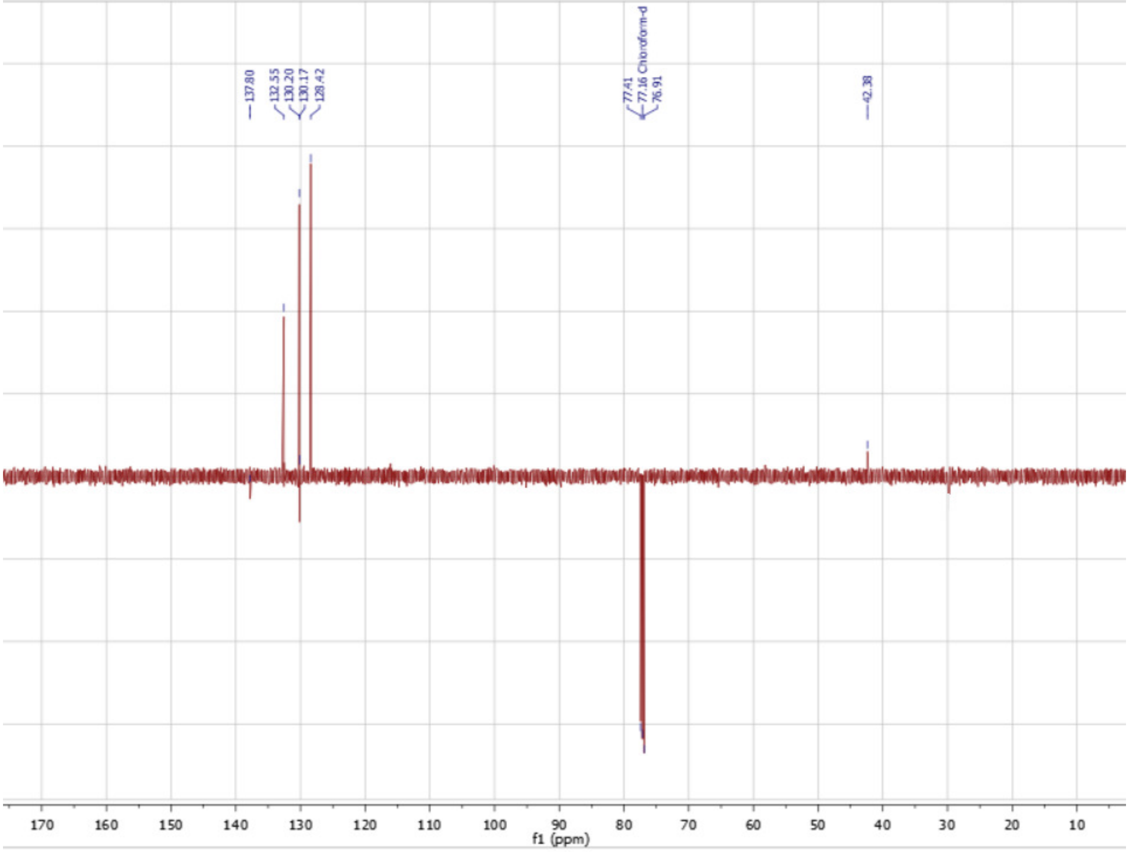

Figure S14. ^{13}C NMR **4f**.

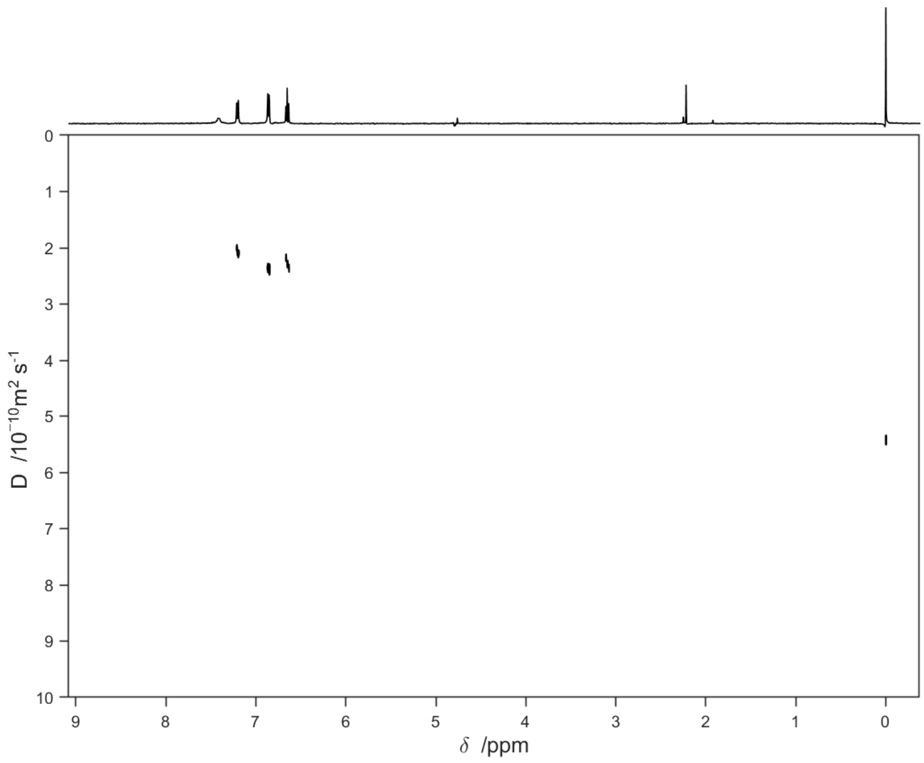

Figure S15. DOSY spectrum of **1**.

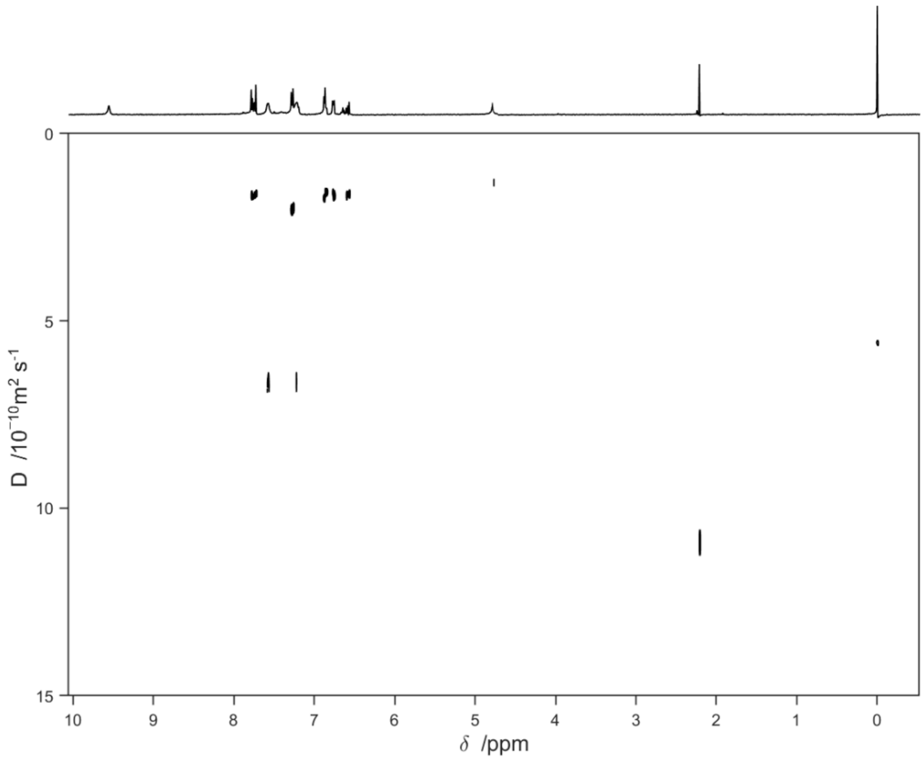

Figure S16. DOSY spectrum of **2a**.

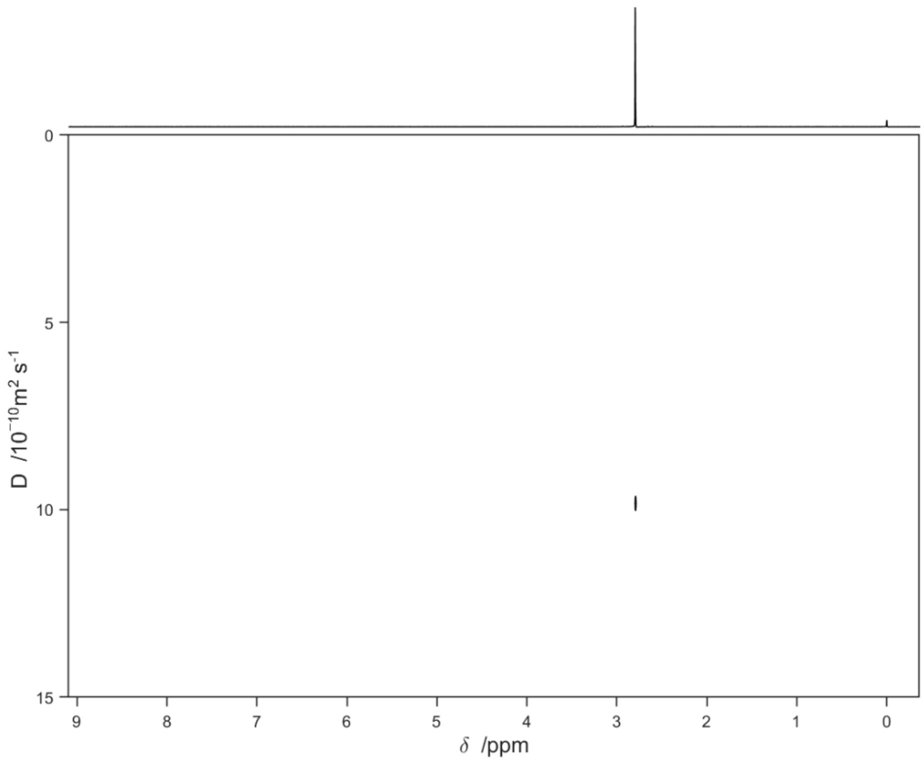

Figure S17. DOSY spectrum of **3**.

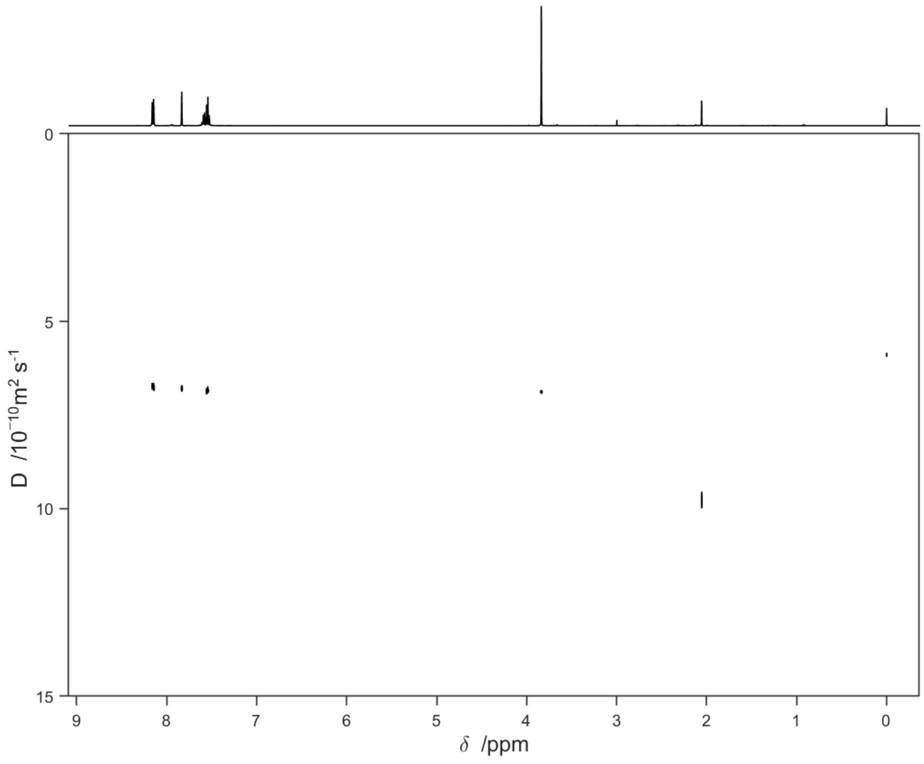

Figure S18. DOSY spectrum of **4a**.

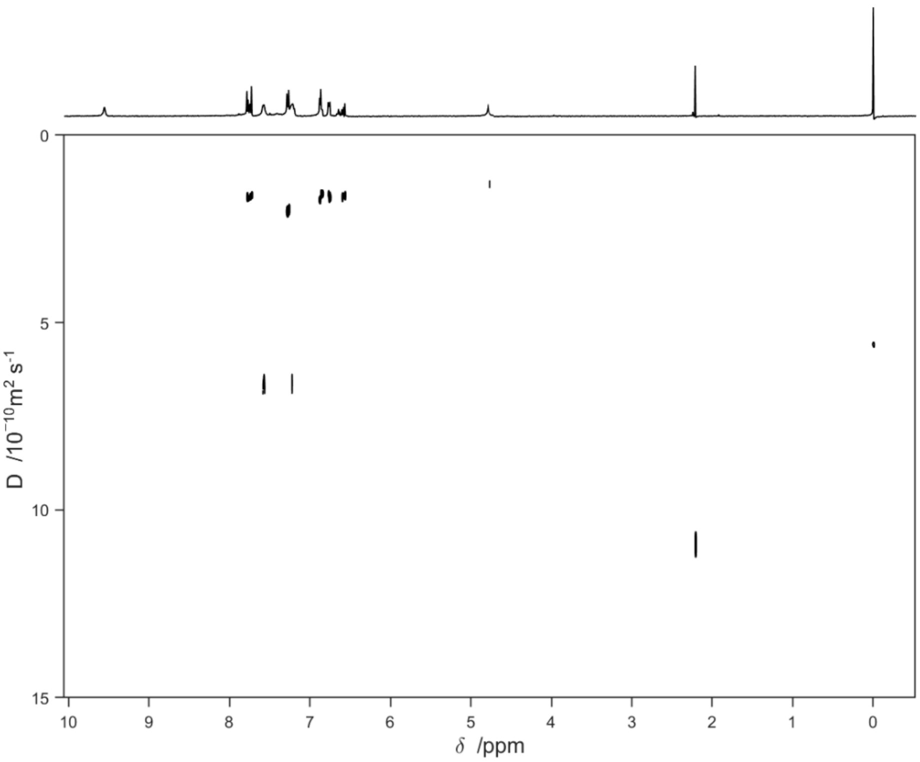

Figure S19. DOSY spectrum of **2a@1**.

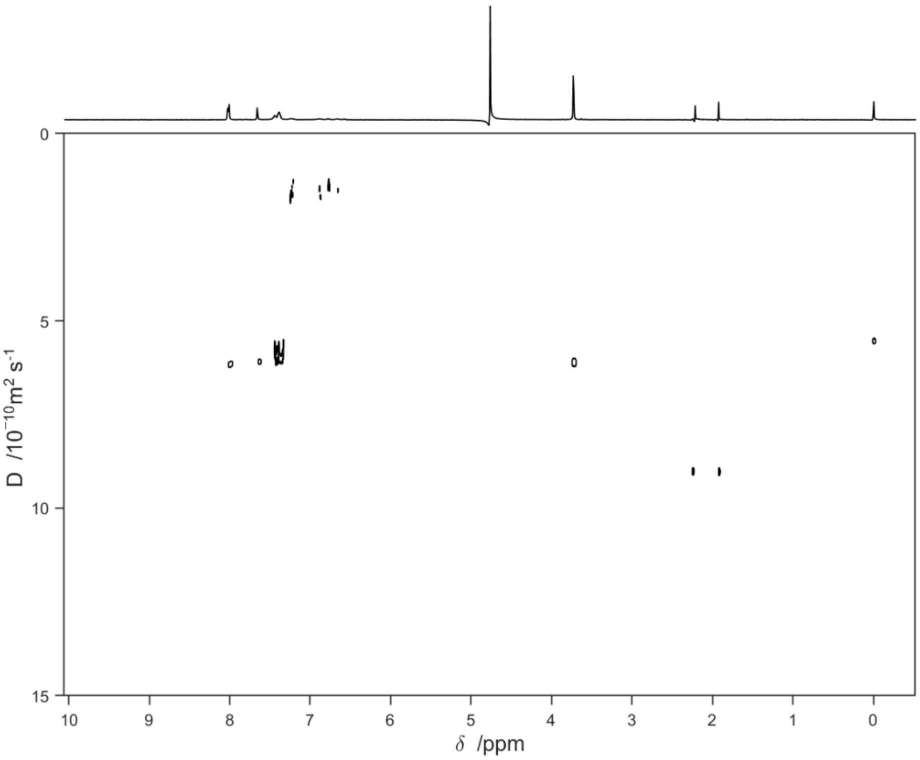

Figure S20. DOSY spectrum of **4a@1**.

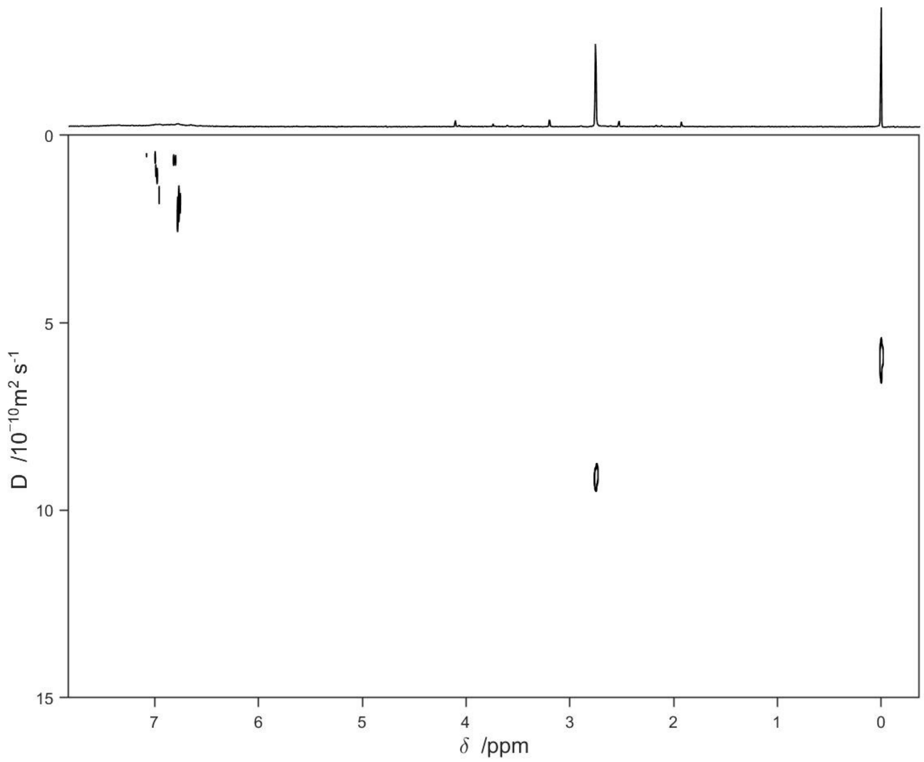

Figure S21. DOSY spectrum of **3@1**.
